# Electrical impedance tomography (EIT) for quantification of pulmonary edema in acute lung injury

**DOI:** 10.1186/s13054-015-1173-5

**Published:** 2016-01-22

**Authors:** Constantin J. C. Trepte, Charles R. Phillips, Josep Solà, Andy Adler, Sebastian A. Haas, Michael Rapin, Stephan H. Böhm, Daniel A. Reuter

**Affiliations:** 1 Department of Anaesthesiology, Center for Anaesthesiology and Intensive Care Medicine, University Medical Center Hamburg-Eppendorf, Martinistrasse 52, D-20246 Hamburg, Germany; 2 Division of Pulmonary and Critical Care Medicine, Department of Medicine, Center for Intensive Care Research, Oregon Health & Science University, Portland, OR USA; 3 CSEM Centre Suisse d’Electronique et de Microtechnique SA, Neuchâtel, Switzerland; 4 Systems and Computer Engineering, Carleton University, Ottawa, ON Canada; 5 Swisstom AG, Landquart, Switzerland

## Abstract

**Background:**

Assessment of pulmonary edema is a key factor in monitoring and guidance of therapy in critically ill patients. To date, methods available at the bedside for estimating the physiologic correlate of pulmonary edema, extravascular lung water, often are unreliable or require invasive measurements. The aim of the present study was to develop a novel approach to reliably assess extravascular lung water by making use of the functional imaging capabilities of electrical impedance tomography.

**Methods:**

Thirty domestic pigs were anesthetized and randomized to three different groups. Group 1 was a sham group with no lung injury. Group 2 had acute lung injury induced by saline lavage. Group 3 had vascular lung injury induced by intravenous injection of oleic acid. A novel, noninvasive technique using changes in thoracic electrical impedance with lateral body rotation was used to measure a new metric, the lung water ratio_EIT_, which reflects total extravascular lung water. The lung water ratio_EIT_ was compared with postmortem gravimetric lung water analysis and transcardiopulmonary thermodilution measurements.

**Results:**

A significant correlation was found between extravascular lung water as measured by postmortem gravimetric analysis and electrical impedance tomography (*r* = 0.80; *p* < 0.05). Significant changes after lung injury were found in groups 2 and 3 in extravascular lung water derived from transcardiopulmonary thermodilution as well as in measurements derived by lung water ratio_EIT_.

**Conclusions:**

Extravascular lung water could be determined noninvasively by assessing characteristic changes observed on electrical impedance tomograms during lateral body rotation. The novel lung water ratio_EIT_ holds promise to become a noninvasive bedside measure of pulmonary edema.

## Background

Assessing a patient’s pulmonary edema is a key factor for guidance of therapy, particularly in critically ill patients. Acute lung injury is multifactorial and often summoned in the clinical definition of acute respiratory distress syndrome (ARDS) [1, 2]. The most common cause of acute lung injury and ARDS is systemic inflammation [3]. The pathophysiologic pattern is characterized by an unspecific inflammatory reaction that results in increased pulmonary vascular permeability. This leads to accumulation of extravascular fluid, formation of edema, and loss of ventilated lung tissue [4]. The physiologic correlate of pulmonary edema is often referred to as *extravascular lung water* (EVLW).

The widely used standard chest x-ray is unreliable for estimation of pulmonary fluid content [5, 6]. X-ray is capable of detecting only changes in EVLW exceeding 38 % [7] and therefore is not suited to guide therapeutic interventions aimed at changing EVLW [8]. A computed tomographic scan can be used to reliably estimate pulmonary fluid content [9, 10] but is not available at the bedside. Therefore, it is associated with a transport trauma of the critically ill patient [11] as well as a radiation exposure that surmounts that of a standard chest x-ray by 100- to 400-fold [12]. Lately, transcardiopulmonary thermodilution (TCPTD) has become a clinical standard for quantifying EVLW. The methodology is available for repetitive bedside use and qualifies as a monitoring tool. Its reliability and validity have been extensively investigated [13–15], and it could be shown to reliably track changes of EVLW exceeding 10 % [16]. Guiding therapy based on EVLW determined by TCPTD was demonstrated to improve patient outcome and to detect fluid overload early in the course of the disease [16, 17]. However, TCPTD still requires invasive catheterization. Particularly, central venous catheters are among the most common causes of nosocomial infection and systemic inflammation [18]. This emphasizes the need to find alternative methods for noninvasive quantification of pulmonary edema. For scientific purposes, postmortem gravimetry still represents the experimental gold standard for estimating pulmonary edema [13, 19–22].

Electrical impedance tomography (EIT) is a noninvasive, radiation-free imaging technology that can be used for various purposes [23, 24]. To date, functional EIT has been investigated mostly to visualize ventilation and to optimize ventilatory therapy in critically ill patients [25–27]. So far, only a single attempt to quantify pulmonary edema by means of EIT has been described, in 1999 [28]. In a pilot experimental series initially intended to confirm these findings, our group failed to reproduce these results (CJ Trepte, J Sola, A Adler, CR Phillips, SH Böhm, DA Reuter). The approach to calculate a novel parameter (lung water ratio_EIT_) is based on the assumption that EVLW fills the dependent parts of the lungs, pushing the ventilation to the nondependent areas of the lung. These changes can be monitored by EIT. Moreover, changes in position will produce a gravity-dependent redistribution of EVLW. These position-related ventilation distribution changes are intended to distinguish between EVLW and other fluid content in the chest and to improve the validity of lung water ratio_EIT_.

Therefore, the aim of the present study was to develop and calculate a novel EIT-based method to estimate pulmonary edema (lung water ratio_EIT_) in acute experimental lung injury and compare it with a gold standard measurement for EVLW using postmortem gravimetry.

## Methods

This study with 30 anesthetized female domestic pigs was designed as a prospective randomized trial. The study was approved by the local governmental commission for the care and use of animals (Institutional Animal Care and Use Committee, Oregon Health & Science University, Portland, OR, USA). This project was part of a larger experimental protocol. A second part of the protocol was dedicated to an independent scientific question addressing the quantification of heart–lung interactions. The animals included in this study received care in compliance with the Guide for the Care and Use of Laboratory Animals (National Institutes of Health publication 86-23, revised 1996), and experiments were conducted in accordance with the Animal Research: Reporting of In Vivo Experiments guidelines [29].

### Anesthesia and instrumentation

The animals were fasted overnight. Premedication was performed with intramuscular injection of ketamine (10 mg kg^−1^), azaperone (4 mg kg^−1^), midazolam (0.5 mg kg^−1^), and atropine sulfate (1 mg). Intravenous access was established, and anesthesia was maintained by continuous infusion of fentanyl (0.05 mg kg^−1^ h^−1^) and propofol (10 mg kg^−1^ h^−1^).

Tracheotomy and placement of an endotracheal tube (8.5 mm) were performed. The animals were monitored with five-lead electrocardiography and pulse oximetry. Volume-controlled mechanical ventilation at an inspiratory oxygen level of 100 % was delivered using a tidal volume (V_T_) of 8 ml kg^−1^, an inspiration/expiration ratio of 1:1.6, and a positive end-expiratory pressure of 10 cmH_2_O. The respiratory rate was kept constant at 18 breaths per minute. Saline was infused at a rate of 13 ml kg^−1^ h^−1^ to maintain hydration.

For catheter placement and surgical preparation, animals were placed in supine position. An 8.5-French central venous catheter was introduced into the right internal jugular vein. Finally, a 5-French thermistor-tipped catheter (item number PVPK2015L20; Pulsion Medical Systems, Feldkirchen, Germany) was placed into the femoral artery for detection of TCPTD. Body temperature was measured using the arterial catheter and kept constant using warming blankets and prewarmed infusions if required.

An EIT belt consisting of 32 electrodes was placed around the thorax, and EIT images were acquired at 50 per second using the ENLIGHT® EIT technology (Timpel, Sao Paulo, Brazil). Meticulous attention was paid to correct placement and sufficient electrical contact of all electrodes. Finally, the animals were wrapped in a vacuum mattress to prevent EIT electrode dislocation throughout the course of the experiments.

### Experimental protocol

The animals were randomized to three different experimental groups. Group 1 served as a sham group and was not subjected to lung injury. In group 2, a direct lung injury was induced by repeated bronchoalveolar lavage [30]. The principle of this method is the depletion and diminution of surfactant factor in the alveoli [30, 31]. This increases the probability of alveolar collapse and results in a decrease of pulmonary compliance. Moreover, surfactant washout has a direct effect on pulmonary defense mechanisms [32, 33]. In group 3, an indirect lung injury via the vascular bed was induced by intravenous application of oleic acid [34]. Oleic acid has a direct toxic effect on endothelial cells, causing epithelial injury with swelling and formation of necrosis [32, 35]. As a result, pulmonary microvascular permeability is markedly increased and leakage of protein rich fluid into the interstitium occurs.

Baseline measurements were performed in all healthy animals and executed with the animals in three distinct positions. Animals were rotated laterally along their longitudinal axis to induce a gravity-dependent redistribution of pulmonary edema (in the supine position, at a 45-degreee left tilt and at a 45-degree right tilt). After changes to each of the defined positions, 20 minutes were allowed for equilibration before data were acquired.

After baseline measurements, lung injury was induced during 1 h. In group 1 (sham), standardized ventilation was continued and no lung injury was induced. In group 2 (lavage), direct lung injury was induced by repeated bronchoalveolar lavage using saline infusion. In group 3 (oleic acid), repeated administration of oleic acid was performed until a total dosage of 0.1 ml kg^−1^ was accomplished as described in previous studies [19, 36, 37].

Three hours after induction of pulmonary injury, continued ventilation measurements were repeated with the animals in all body positions. Immediately after completion of data acquisition, the animals were killed during deep anesthesia and their lungs were removed for postmortem gravimetric analysis of EVLW as described by Rossi et al. [13].

### Data collection

With each point of measurement—that is, each body position—EIT data were recorded for 120 seconds and a triplicate injection of a cold bolus (15 ml of saline infusion) was performed for estimation of EVLW from TCPTD. Ventilatory data were recorded from the respirator (AVEA®; CareFusion, San Diego, CA, USA). Arterial blood gas analysis was performed in baseline and injured lung conditions. Gravimetric analysis was executed at the end of the protocol.

### Data processing of EIT data and calculation of lung water ratio_EIT_

**Fig. 1:**
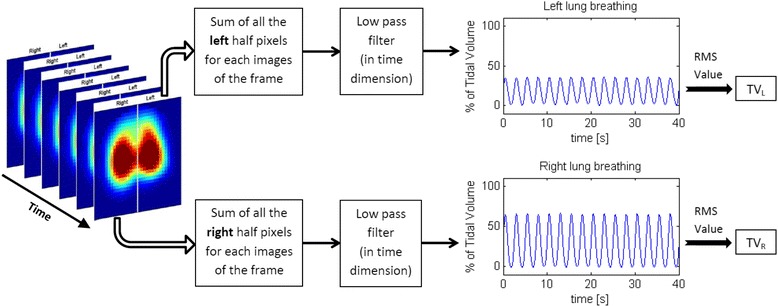
Process for extracting the tidal ventilation of the left lung and the tidal ventilation of the right lung (TV_L_ and TV_R_, respectively) from sets of electrical impedance tomographic images. *RMS* root mean square

Each EIT dataset was processed offline. The EIT-based parameter lung water ratio_EIT_ was calculated from data in the three defined body positions. First, the differences in tidal ventilation (TV) between the left and the right lungs were calculated. For this purpose, the sum of the TV signals of all pixels representing the left and right hemithorax were computed separately and filtered using a Butterworth low-pass filter (cutoff frequency 2 Hz, filter order 8) to remove higher-frequency noise and cardiac activity. TV within the left and right hemithorax was expressed as the percentage of the total TV of both lungs. Two coefficients were computed by taking the root mean square value of the cyclic components of the two aforementioned V_T_ signals. These coefficients were then labeled TV_L_ and TV_R_ and represented the tidal ventilation within the left and right lungs, respectively (Fig. 1). From these two coefficients, an imbalance coefficient (IM_L–R_) was calculated that expressed the ratio of ventilation between the left and right lungs:

$$ I{M}_{L-R}={\scriptscriptstyle \frac{T{V}_L-T{V}_R}{T{V}_L+T{V}_R}} $$

This parameter was normalized between +1 (only the left lung is ventilated; TV_R_ = 0) and −1 (only the right lung is ventilated; TV_L_ = 0). This imbalance coefficient (IM_L–R_) was calculated for each pig in each position at baseline and after acute lung injury.

**Fig. 2:**
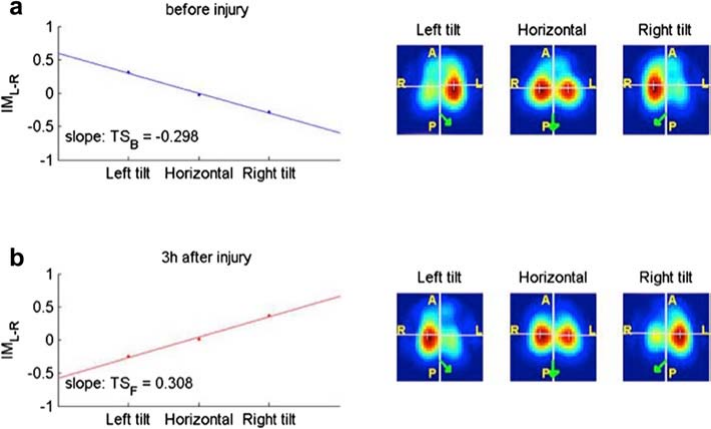
Calculation of lung water ratio_EIT_: Trend slopes (TS) computed from the imbalance coefficient (IM_L–R_) of each pig in the three different body positions of the protocol. **a** Conditions in healthy lungs. **b** Conditions in injured lungs

For calculation of lung water ratio_EIT_, a set of data collected at the three different lateral rotation angles during baseline and acute lung injury was used to calculate a characteristic slope (Fig. 2). The parameter lung water ratio_EIT_ is the slope of the trend line (least square regression, first order) assuming that the distance on the *x*-axis between two successive positions (left tilt, horizontal and right tilt) is 1.

### Statistics

Statistical analysis was performed using SigmaPlot 12.0 software (Systat Software, San Jose, CA, USA). Tests for normality were carried out using the Kolmogorov-Smirnov test. The results are presented as mean ± standard deviation. Comparison of the different study groups and analysis of effects of group and treatment interaction were performed using two-way analysis of variance. Effects of pulmonary injury within the groups were analyzed using paired *t* tests. Differences between the groups after experimental lung injury were evaluated using unpaired *t* tests. The strength of coherence between lung water ratio_EIT_ and EVLW from postmortem gravimetry was evaluated using Pearson product-moment correlation. Statistical significance was set at *p* < 0.05.

## Results

### Animal characteristics

The protocol was completed in 29 animals, comprising 8 in group 1 (sham), 11 in group 2 (oleic acid), and 10 in group 3 (lavage). One animal in the lavage group was lost after induction of pulmonary injury and was excluded from further statistical analysis. Mean body weight and length were, respectively, 34.5 ± 3.2 kg and 105.3 ± 4.6 cm in group 1 (sham), 34.0 ± 1.8 kg and 104.3 ± 2.4 cm in group 2 (lavage), and 34.2 ± 2.6 kg and 105.7 ± 3.7 cm in group 3 (oleic acid). No statistical difference between the groups regarding body weight or length was found.

### Ventilation and pulmonary function

Details regarding the effects of lung injury on ventilation, as well as the results of blood gas analysis, can be found in Table 1.

**Table 1:** Ventilation data before and after induction of experimental lung injury

	pAWP (cmH_2_O)	mAWP (cmH_2_O)	dc (ml/cmH_2_O)	paO_2_ (mmHg)
Baseline				
Group 1 (sham)	22.8 ± 3.7	14.2 ± 1.0	23.3 ± 5.1	551 ± 63
Group 2 (lavage)	27.7 ± 7.2	16.1 ± 3.0	18.2 ± 6.5	524 ± 68
Group 3 (oleic acid)	23.1 ± 2.8	14.3 ± 1.3	21.4 ± 3.8	507 ± 95
Lung injury				
Group 1 (sham)	21.9 ± 2.3	13.9 ± 0.8	24.4 ± 5.1	527 ± 122
Group 2 (lavage)	35.5 ± 7.1^a^	17.7 ± 3.3	11.8 ± 2.5^a^	186 ± 155^a^
Group 3 (oleic acid)	35.0 ± 5.4^a^	17.1 ± 1.9^a^	11.5 ± 2.3^a^	146 ± 116^a^

*pAWP* on peak airway pressure, *mAWP* mean airway pressure, *dc* dynamic respiratory system compliance, *paO*_*2*_ arterial partial pressure of oxygen

Data are presented as mean ± standard deviation

^a^Statistically significant differences between baseline and lung injury (*p* < 0.05)

### Lung water ratio_EIT_

**Fig. 3:**
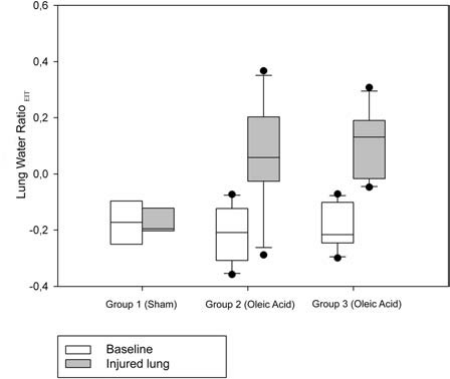
Box plots for lung water ratio_EIT_. Data for the three study groups before and after induction of experimental lung injury are presented. *Statistically significant difference compared with baseline before lung injury (*p* < 0.05)

Intergroup comparison did not reveal significant differences at baseline. In the sham group animals, lung water ratio_EIT_ did not change significantly along the protocol (−0.127 ± 0.184 to −0.170 ± 0.054). In contrast, lavage and oleic acid infusion resulted in statistically significant changes in lung water ratio_EIT_, from −0.209 ± 0.098 to 0.0679 ± 0.174 and from −0.189 ± 0.078 to 0.110 ± 0.120, respectively (Fig. 3 and Table 2).

**Table 2:** Changes of lung water ratio_EIT_ with induction of acute lung injury

Group	Baseline lung water ratio_EIT_	Injury lung water ratio_EIT_
Group 1 (sham)	−0.127 ± 0.184	−0.170 ± 0.054
Group 2 (lavage)	−0.209 ± 0.098	0.0679 ± 0.174^a^
Group 3 (oleic acid)	−0.189 ± 0.078	0.110 ± 0.120^a^

Data are presented as mean ± standard deviation

^a^Statistically significant differences between baseline and lung injury (*p* < 0.05)

### Transcardiopulmonary thermodilution

EVLW_TD_ from TCPTD showed the same pattern as lung water ratio_EIT_ (Table 3).

**Table 3:** Extravascular lung water by transcardiopulmonary thermodilution

	EVLW_TD_ (ml m^−2^)	
Group	Baseline	Injury
Group 1 (sham)	327.0 ± 86.4	346.3 ± 55.6
Group 2 (lavage)	316.7 ± 32.6	630.4 ± 179.6^a^
Group 3 (oleic acid)	366.7 ± 167.7	488.3 ± 173.8^a^

*EVLW*_*TD*_ extravascular lungwater measured by transcardiopulmonary thermodilution

^a^Statistically significant difference between baseline and lung injury (*p* < 0.05)

### Correlation analysis of lung water ratio_EIT_ and postmortem gravimetry

**Fig. 4:**
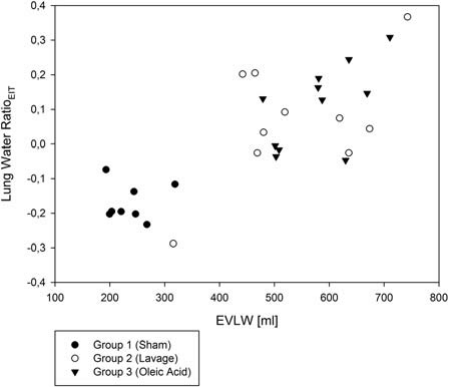
Correlation of lung water ratio_EIT_ after induction of experimental lung injury and extravascular lung water (EVLW) by postmortem gravimetry

Correlation of lung water ratio_EIT_ and EVLW from postmortem gravimetry was strong (*r* = 0.80; *p* < 0.001) (Fig. 4).

## Discussion

This study demonstrates that EIT can be used for noninvasive quantification of pulmonary edema in acute experimental lung injury. The development of lung water ratio_EIT_ represents an approach to assessment of EVLW as a physiologic correlate of pulmonary edema at the bedside, as well as to differentiation between healthy and injured lungs.

Assessing pulmonary edema by EIT has the methodological challenge that, at present, EIT can be used only to quantify changes and not to measure absolute values [27, 38]. In absolute EIT measurements, positioning of the electrodes, dimensions and shape of the body, and differences in individual skin conductance do affect signal analysis. This becomes even more difficult when tissue properties change, such as in pulmonary edema, and the differentiation between the myriad of different influencing factors is not yet possible. These issues can be circumvented by applying functional EIT imaging of the lungs because these factors only slightly differ between inspiration and expiration. By subtracting from each EIT image a respective reference image, most inaccuracies can be eliminated, ultimately producing EIT images that reflect nothing but the changing lung conditions in question. Earlier experiments demonstrated that functional EIT was able to detect changes in impedance behavior due to lung injury during ventilation. Frerichs et al. demonstrated that disturbances in lung function and a redistribution of ventilation can be followed by EIT in an experimental model that also used oleic acid to induce experimental lung injury [39]. Using another approach, Adler et al. reported that EIT was able to demonstrate a decrease in ventilation after pulmonary fluid instillation [40]. However, a direct quantification of pulmonary edema did not seem possible. For this reason, a different approach was necessary, based on the rationale that the distribution of ventilation will change within the edematous lung and that, owing to gravity, fluid will accumulate in the dependent parts. A similar approach was first described by Kunst et al. in an experimental series of 14 patients with ARDS [28]. They demonstrated that occurrence of ARDS resulted in characteristic changes of ventilatory distribution. In this approach, patients were positioned in supine position and impedance changes in the nondependent, anterior half of the thorax were divided by the mean value of impedance changes of all pixels. Surprisingly, the results of this study showed that impedance changes—representing ventilatory changes—were shifted toward the dependent, dorsal parts of the lungs with ARDS. These counterintuitive results could be reproduced neither in our pilot experiments nor in our present study. Also, Czaplik et al. demonstrated that induction of acute lung injury and pulmonary edema by intravascular injection of oleic acid resulted in a regional reduction of impedance changes in the dependent, dorsal areas of the lung, as well as reduced air content in the corresponding alveoli, by using in vivo confocal laser endomicroscopy [41]. These findings are further supported by those of Fagerberg et al., who used EIT to show that ventilation in the posterior, dependent areas of the lung decreased with experimental lung injury induced by *Escherichia coli* lipopolysaccharide, shifting ventilatory changes toward the anterior, nondependent areas of the lung [42].

The rationale for our methodology was to use an intervention (small rotations of the subject) to shift EVLW. Our hypothesis was that if ventilatory distribution is evaluated in each individual position, it would help to characterize the individual lung condition and potentially enable us to differentiate redistributable pulmonary edema from other altered ventilatory conditions. That means that an inflammatory infiltrate might also result in changes of the distribution pattern of ventilation; however, according to our hypothesis, changes would not be gravity-dependent and redistribute with different body positions. When we analyzed the strength of association between the noninvasive lung water ratio_EIT_ and EVLW from postmortem gravimetry, we found a highly significant correlation. Unfortunately, the results of our study do not allow us to present further statistical analysis for assessing methodological agreement. While Lung water Ratio_EIT_ analysis changes in the distribution pattern of ventilation that occurs with presence of pulmonary edema, gravimetric analysis exactly quantifies the amount of pulmonary fluid content. Therefore, both methods will produce differing results related to the quantity of pulmonary edema, but they do not measure exactly the same thing. Of particular note is that both lung water ratio_EIT_ and gravimetric analysis of EVLW were able to separate healthy from injured lung conditions. In our experimental series, we found only one outlier, an animal of the lavage group, which presented with normal EVLW values in both postmortem gravimetry and lung water ratio_EIT_. The explanation appears to be that, in this animal, repeated bronchoalveolar lavage did not result in a relevant lung injury. Neither gravimetric measurements nor lung water ratio_EIT_ were able to discriminate between the two different models of acute lung injury. In the future, discrimination of cardiogenic and noncardiogenic edema for lung water ratio_EIT_ is conceivable, which could be based either on differing distribution patterns of ventilation or on differences in conductivity.

The impairment of oxygenation in our study was documented by relevant decreases in oxygenation ratios in both injury groups corresponding to moderate ARDS [1, 3]. Currently, we cannot present a specific cutoff value for lung water ratio_EIT_ that identifies the beginning of lung injury and pulmonary edema. Future work is needed to determine the sensitivity with which lung water ratio_EIT_ can reliably discriminate healthy from less severely injured lungs. Despite the fact that the amount of pulmonary edema can be determined only one time by the experimental gold standard of postmortem gravimetry, the idea that lung water ratio_EIT_ changes with the onset of acute lung injury and pulmonary edema is backed by the results obtained from TCPTD. The fact that EVLW_TD_ increases with the induction of experimental lung injury supports the validity of our concept. Even though EVLW_TD_ and lung water ratio_EIT_ point in the same direction, it is too early to determine the role of lung water ratio_EIT_ to reliably trace changes of EVLW over time.

Another aspect is that currently we do not know whether and to what extent lung water ratio_EIT_ will be influenced by pulmonary infections or inflammatory infiltrates and whether the differentiation of pulmonary edema from an actual infection will be possible. Therefore, our study has to be considered a feasibility study. The proof of our hypothesis that lung water ratio_EIT_ is characteristic for the occurrence of pulmonary edema and capable of differentiating pulmonary edema from other effects on the lung will require further dedicated studies.

The models of acute lung injury we used in our study are both well established. However, neither repeated bronchoalveolar lavage nor intravascular administration of oleic acid can totally mimic lung injury and pulmonary edema as they occur in clinical practice. Loss of surfactant factor as induced by repeated alveolar lavage is a key element in ARDS; however, it is a consequence rather than the main cause of lung injury and best corresponds to the early phase of ARDS. A key methodological weakness of this model is its lack of standardization of the induced lung injury, as can also be seen in our results. This is different from oleic acid infusions, which are particularly reproducible and easily standardized. Basically, this model mimics a massive fat embolism, which admittedly is not the most common cause of clinical ARDS. A further aspect is that both models will result in relevant effects of ventilation–perfusion matching [43]. This is also true in a clinical situation, but from a methodological aspect is particularly relevant for TCPDT because changes in the ventilation/perfusion ratio and occurrence of perfusion defects due to microembolization are associated with changes in EVLW obtained by TCPTD. As a future perspective, this might be another methodological advantage of estimating pulmonary edema by means of EIT because this technology also allows measurement of pulmonary perfusion [44–46].

## Conclusions

EIT enabled noninvasive determination of EVLW and discrimination of healthy and injured lung conditions in our experimental model of acute experimental lung injury. The novel lung water ratio_EIT_ holds promise to become a noninvasive bedside measure of pulmonary edema.

## Key messages

- Estimation of pulmonary edema is vital for monitoring and guidance of therapy in critically ill patients.
- Electrical impedance tomography (EIT) allows determination and differentiation of healthy and injured lung conditions.
- The novel parameter lung water ratio_EIT_ enables quantification of pulmonary edema by EIT.
- EIT holds the potential to become a reliable, noninvasive technology with which to monitor pulmonary edema at bedside.

## Abbreviations

ARDS: acute respiratory distress syndrome
dc: dynamic respiratory system compliance
EIT: electrical impedance tomography
EVLW: extravascular lung water
IM_L–R_: imbalance coefficient
mAWP: mean airway pressure
paO_2_: arterial partial pressure of oxygen
pAWP: peak airway pressure
TCPTD: transcardiopulmonary thermodilution
V_T_: tidal volume
